# Deep Reinforcement Learning Based Decision Making for Complex Jamming Waveforms

**DOI:** 10.3390/e24101441

**Published:** 2022-10-10

**Authors:** Yuting Xu, Chao Wang, Jiakai Liang, Keqiang Yue, Wenjun Li, Shilian Zheng, Zhijin Zhao

**Affiliations:** 1 Key Laboratory of RF Circuits and Systems, Ministry of Education, Hangzhou Dianzi University, Hangzhou 310018, China; 2 Science and Technology on Communication Information Security Control Laboratory, The No. 011 Research Center, Jiaxing 314033, China; 3 The School of Communication Engineering, Hangzhou Dianzi University, Hangzhou 310018, China

**Keywords:** cognitive radio, intelligent jamming, deep reinforcement learning, Wolpertinger architecture, soft actor-critic

## Abstract

With the development of artificial intelligence, intelligent communication jamming decision making is an important research direction of cognitive electronic warfare. In this paper, we consider a complex intelligent jamming decision scenario in which both communication parties choose to adjust physical layer parameters to avoid jamming in a non-cooperative scenario and the jammer achieves accurate jamming by interacting with the environment. However, when the situation becomes complex and large in number, traditional reinforcement learning suffers from the problems of failure to converge and a high number of interactions, which are fatal and unrealistic in a real warfare environment. To solve this problem, we propose a deep reinforcement learning based and maximum-entropy-based soft actor-critic (SAC) algorithm. In the proposed algorithm, we add an improved Wolpertinger architecture to the original SAC algorithm in order to reduce the number of interactions and improve the accuracy of the algorithm. The results show that the proposed algorithm shows excellent performance in various scenarios of jamming and achieves accurate, fast, and continuous jamming for both sides of the communication.

## 1. Introduction

As a key link in cognitive electronic warfare, cognitive electronic jamming decisions are susceptible to adversarial attacks, such as spoofing attacks or mimicry attacks, because of the inherent openness of the wireless medium [1]. In these cases, attackers can claim to be real users by imitating them, especially in device-to-device (D2D)-dependent loosely distributed infrastructures [2]. The emergence of various anti-jamming communication techniques to ensure secure transmission, such as frequency modulation (FM) technology [3], burst communication technology [4], and cognitive radio technology [5], increases the difficulties of jamming techniques. Therefore, the study of cognitive jamming decision methods is crucial.

Traditional jamming methods, including continuous jamming [6,7,8], reactive jamming [9,10], spoofing jamming [11,12], random periodic jamming [10], sweeping jamming [13], etc. [14], cannot perform accurate jamming or efficiently utilize jamming resources in complex battlefield communication environments.

As an important branch of mathematics, various metaheuristics algorithms are widely used in optimization problems and achieve excellent performance. For example, the hybrid sine and cosine algorithm combined with the fuzzy k-nearest neighbor method (SCA FKNN) proposed in [15] has achieved good accuracy in classification and prediction results with 10 different types of data sets. The clonal selection algorithm (CSA) and logic mining approach were used to solve the problem of Amazon employee resource access data extraction [16].The binary artificial bee colony method was used to optimize non-systematic weighted satisfiability [17]. Similarly, various intelligent optimization theories have been applied to the field of wireless communication jamming. Q., Z. et al. ran game theory separately on cognitive radio networks and cognitive jammers and the Nash equilibrium became the final regularly chosen behavior [18]. Fang Ye et al. proposed a taboo search artificial bee colony (TSABC) algorithm for the cognitive cooperative jamming decision, which performed better than the improved ant colony (IAC) algorithm and the artificial bee colony (ABC) algorithm, with better search capability and probability [19]. Intelligent optimization theory often requires known channel and signal priori information. However, it is difficult for the jammer to obtain this information in a battlefield environment.

Reinforcement learning (RL) has received a lot of attention in recent years as a branch of machine learning that has reached and even surpassed human levels in Go and video games. Reinforcement learning is an intelligent agent that optimizes its own action strategy through the acquisition of reinforcement signals (reward feedback) from the environment [20]. Prior information is not necessary to the agent, and the training data of reinforcement learning come from the continuous interaction with the environment. Because of these characteristics, reinforcement learning has become a powerful tool for resource optimization, jamming decisions, and other areas of intelligent jamming. Meanwhile, deep reinforcement learning (DRL) [21] technology has been employed to solve non-convex optimization problems in communication systems [22]. The development of artificial intelligence technology has provided new solutions to cognitive jamming technology [23,24]. Amuru et al. [1] used the multi-armed bandit (MAB) [25] model to develop a cognitive jammer in unknown battlefield environments, where the jammer can select physical layer parameters for flexible jamming. Zhuan Sun, S. et al. [26] combined the advantages of orthogonal matching tracking (OMP) [27] and MAB to significantly reduce the number of interactions. However, the MAB model mainly targets the static transmitter-receiver and requires re-learning for dynamic targets with changing parameters. Yangyang Li et al. [28] designed an intelligent jamming method based on reinforcement learning to combat the DRL-based user and experimentally demonstrated that the proposed algorithm can effectively limit the performance of the DRL-based anti-jamming method, which targets only the physical layer parameter of the spectrum. In [29], a multi-intelligent reinforcement learning framework with an optimal power control strategy in a dynamic game between smart jammers and training base stations was designed, and experiments showed that smart jammers with eavesdropping capabilities can seriously degrade the performance of the jammed communication parties.

When the communication parties choose to adjust the communication parameters after being jammed, the situation faced by the jammer becomes complex, i.e., the problem of a large-scale discrete action space in reinforcement learning develops. To our knowledge, there are few studies on intelligent jamming decisions in large action spaces. When the number of action spaces is very large, a straightforward idea is to serialize discrete actions and then use reinforcement learning algorithms that solve continuous actions to solve large-scale discrete action problems. In solving the problem of large-scale discrete actions in reinforcement learning, the DeepMind team proposed the Wolpertinger architecture in 2015 [30], and the authors validated the superiority of the proposed algorithm on three environments using the deep deterministic policy gradient (DDPG) algorithm in cart-pole, puddle world, and recommender systems; Haokun Chen et al. proposed a tree-structured policy gradient recommendation (TPGR) framework in 2019 [31], where a balanced hierarchical clustering tree is built over the items and picking an item is formulated as seeking a path from the root to a certain leaf of the tree.

The case considered in this paper is as follows: We investigate a soft actor-critic [32] optimization for an intelligent jamming waveform decision system. A complex jamming scenario in which communication parties choose to adjust the physical layer parameters in order to avoid jamming is considered. In particular, the jammer learns the anti-jamming strategy for both sides of the communication procedure with as few interactions as possible in the face of a complex jamming waveform decision situation. We apply the SAC technique to solve the considered problem that occupies continuous and large-dimensional optimization variables. The contributions of the paper are mainly as follows:

- A DRL-based algorithm to optimize intelligent jamming waveform decision problems is proposed. The correspondence between the communication state of the communication parties and the optimal jamming policy is established. The proposed DRL-based algorithm is based on the Markov decision process (MDP), which is utilized to deal with the problems of goal-directed learning from interaction.
- To solve the considered problems of slow convergence and the long interaction times of the intelligences in a large-scale discrete action space, we propose a deep reinforcement learning smart jamming decision algorithm based on a maximum-entropy SAC that incorporates the improved Wolpertinger architecture based on the original SAC algorithm. Experiments show that the proposed algorithm has good convergence speed and high jamming accuracy for both small and large jamming action space scenarios. In some scenarios, the jamming success rate can quickly reach 100%. It is worth noting that, to the best of our knowledge, the SAC method has not yet been used in the communication jamming waveform control field.
- To prevent jammers from blindly pursuing high rewards and choosing the highest power series of actions, we design a power penalty factor for the reward. To balance the exploration and exploitation dilemmas in different periods of jamming, we design the dynamic entropy coefficient.

The rest of the paper is organized as follows: In Section 2, we introduce the reinforcement learning algorithm and the intelligent jamming system model. Section 3 presents the improved SAC algorithm and the details of the algorithm. In Section 4, extensive simulation experiments are conducted to verify the performance of the algorithm proposed in this paper and the results are analyzed. Section 5 summarizes the contributions of this paper and discusses some conclusions obtained from this study.

## 2. System Model and Problem Formulation

In this paper, a communication jamming system in a non-cooperative scenario is considered. In the communication process, anti-jamming technologies, such as power enhancement, channel switching, and modulation switching, are adopted to suppress the effects caused by jamming signals. The jamming target in such a scenario is changing over time, making traditional MAB models for static targets and tasks unavailable.

### 2.1. Reinforcement Learning

**Figure 1:**
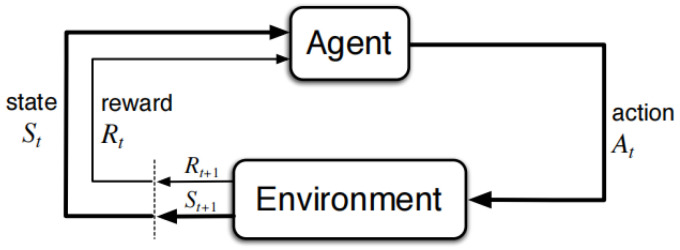
The agent–environment interaction in a Markov decision process.

The essence of reinforcement learning can be described as maximizing the rewards that can be obtained in uncertain environments. It consists of two major elements: the agent and the environment. As shown in Figure 1 [33], the agent interacts with the environment and outputs an action, $a_{t}$, based on the state, $s_{t}$, of the environment, and the environment goes to the next state, $s_{t+1}$, and gives a feedback or reward, $R_{t}$, under the influence of this action. The goal of the agent is to maximize the sum of the rewards, $G_{t}=\overset{\infty }{\underset{k=0}{\sum }}\gamma^{k}R_{t+k+1}$. The discount factor, γ∈(0,1), indicates less attention to the longer-term reward. The state value function can be obtained from the discounted reward $G_{t}$ function, which is used to evaluate the value of a state. In addition, the action state value function $Q_{\pi }\left(s,a\right)=E[G_{t}|s_{t}=s,a_{t}=a]$ is introduced to represent the possible reward for taking a certain action in a certain state.

Reinforcement learning can be divided into policy-based and value-based approaches. In the policy-based approach, the policy $\pi_{\theta }=(a|s)$ is assumed to be a continuous differentiable function with respect to θ. The policy-based approach uses a gradient ascent method to optimize θ to maximize the gradient of the policy, E[G]. In the value-function-based approach, the agent continuously updates the state value function, $Q_{\pi }$, based on feedback and selects the action with the largest Q value as the actual policy. The algorithm used in this paper is based on policy gradients and value-based actor-critic structures, which can solve both the problem of the slow convergence speed of policy-based methods and the inability of value-function-based methods to adapt to high-dimensional or continuous actions.

### 2.2. System Model

**Figure 2:**
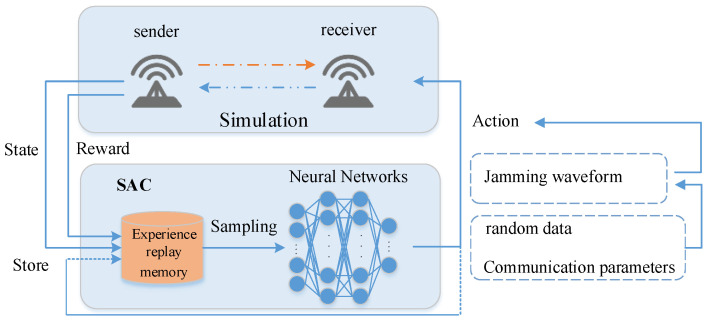
Main components and data flow of SAC algorithm.

A cognitive electronic warfare jamming scenario is considered, in which the communication parameters of the communication parties include modulation $\left\{M_{1},M_{2},\ldots ,M_{n}\right\}$, transmission power $\left\{P_{1},P_{2},\ldots ,P_{m}\right\}$, and communication frequency points $\left\{C_{1},C_{2},\ldots ,C_{l}\right\}$. A combination action of lowering the modulation order, increasing the transmission power, and switching the communication frequency is taken when the communication parties are jammed. The jammer can obtain the signal modulation mode, communication band, and approximate transmit power of the jammed party through communication reconnaissance in the actual jamming. Each jammer in this paper is equipped with a cognitive engine that can reconnoiter some basic communication parameters of the communication parties. The parameters of the jammer are also composed of the modulation mode, jamming power, and jamming frequency. The jammer generates each sample by interacting with the environment and stores it in experience pool *D*. Then, the algorithm is trained by the samples randomly extracted from experience pool *D*. Figure 2 shows the main components and data flow of SAC algorithm based on this system.

**Figure 3:**
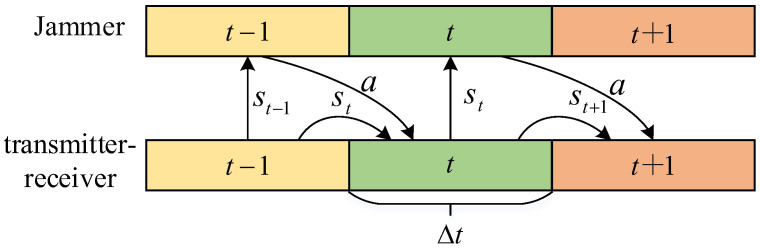
Jamming timing schematic.

In order to conduct accurate and continuous jamming, the jammer needs to learn the complex anti-jamming strategies of the communication parties. The state, $s_{t}$, is composed of the communication state of the communication parties at moment t and the jamming action, $a_{t}$, of the jamming policy to the communication parties at moment t. In addition, the action, $a_{t}$, is the jamming action for the communication parties at the next moment, t+1. The purpose of this design is as follows: Due to the confidentiality of the communication system and the complexity of the electromagnetic environment, the communication parameters of the communication parties are difficult to detect in a short period of time. Even if communication parameters can be detected in a shorter period of time, the jamming effect may be poor due to the insufficient jamming time. Figure 3 shows the jamming timing diagram of this paper.

### 2.3. Reward Function

In reinforcement learning, an agent interacts with and obtains a reward from the environment. The agent updates its policy based on the reward. However, the reward is often difficult to obtain in intelligent jamming scenarios. In [1], the authors used the symbol error rate (SER) as a criterion for the reward, assuming that the communication parties used TCP/IP protocol as a precondition. In this paper, we similarly assume that the jamming party can obtain approximate information about the SER of the communication parties and use it as an evaluation criterion. The reward function is therefore designed as follows:

(1) $$Reward=\{\begin{array}{c} 0.3\times 100+a_{p}\times \left(-1\right)\times 3+60+F_{1}\times 30;\;\mathrm{if}\;\left(\mathrm{SER}\geq X\right)\\ \frac{10}{\left(\mathrm{fabs}\left(s_{c}-a_{c}\right)\right)+1}\times 1.2;\;\mathrm{if}\;\left(\mathrm{SER}<X\right) \end{array}$$

where $a_{p}$ represents the jamming power, $a_{c}$ denotes the jamming frequency, and $s_{c}$ denotes the communication frequency of both communication parties. $F_{1}$ denotes the frequency alignment parameter, which is 1 if the frequency of the jamming action and the frequency of both communication parties are the same; otherwise, it is 0. X denotes the threshold value of the SER. The above equation shows that if the SER is greater than a certain threshold, a higher reward is given, with the addition of a penalty factor and a reward factor. The penalty factor, $a_{p}\times \left(-1\right)\times 3$, is a penalty on the power, which is used for the purpose of preventing the jammer from blindly selecting some series of actions with maximum power. The reward factor, $F_{1}\times 30\;$, represents the prize for channel alignment, which is designed so that channel alignment is a prerequisite for successful jamming. If the SER is less than the threshold, X, the absolute value of the difference between the frequency point of the jamming action and the frequency point of the communication parties is used as the reward criterion, which is designed so that the agent can learn information, even from the experience of jamming failure.

## 3. Proposed Jamming Scheme Based on SAC Model

### 3.1. Introduction of SAC Algorithm

The SAC algorithm is an off-policy approach to optimize stochastic policy. The core idea is maximum entropy reinforcement learning, in which the goal of the agent in the SAC algorithm is to maximize the expected reward and entropy. The introduction of entropy allows the policy to be as random as possible. The goal of standard reinforcement learning is to maximize the reward sum, $\underset{t}{\sum }{Ε}_{\left(s_{t},a_{t}\right)\sim \rho_{\pi }}\left[r\left(s_{t},a_{t}\right)\right]$, where $\rho_{\pi }$ represents the distribution of policy. For in the SAC algorithm, the goal of its optimization is defined as:

(2) $$\sum {Ε}_{\left(s_{t},a_{t}\right)\sim \rho_{\pi }}[r\left(s_{t},a_{t}\right)+\alpha H(\pi (\cdot |s_{t}))]$$

where $H(\pi (\cdot |s_{t}))=-log(\pi (a_{t+1}|s_{t+1}))$ represents the entropy part of the SAC algorithm. $\pi (a_{t+1}|s_{t+1})$ denotes a stochastic policy, π, of selecting an action, $a_{t+1}$, under a state, $s_{t+1}$.The α is the temperature coefficient, which determines the importance of entropy for the reward and controls the randomness of the optimal strategy. It becomes standard reinforcement learning at α=0 [32,34].

Compared with the proximal policy optimization (PPO) [35] reinforcement learning algorithm for online learning, the SAC algorithm follows the experience replay technique in deep Q learning (DQN) [12]. Sample utilization is important in the jamming scenarios mentioned in this paper, where each interaction is a valuable experience, and we hope that the switching strategy of the communication parties can be learned with as few interactions as possible. Compared with deep deterministic policy gradient (DDPG) [36], which is sensitive to hyperparameters and unstable in performance, the SAC algorithm integrates the three major frameworks of actor-critic, off-policy, and the maximum entropy model. The intelligent jamming algorithm sets a larger entropy coefficient in the early stage of jamming to increase the exploration of the environment and gradually reduces it in the later stage to improve the accuracy of jamming. The SAC algorithm not only greatly improves sample utilization but also has fewer hyperparameters. The addition of entropy also makes it insensitive to hyperparameters. The algorithm assigns approximately equal probabilities to actions with similar Q values, avoiding the condition where the agent repeatedly selects actions and falls into suboptimal situations. Experience has shown that the SAC algorithm surpasses other reinforcement learning algorithms in continuous control problems.

The SAC algorithm contains two kinds of networks: the policy network $\pi_{\varphi }(a_{t}|s_{t})$ with parameter φ and the value network $Q_{\theta }\left(s_{t},a_{t}\right)$ with parameter θ. The policy network outputs actions, and the value network evaluates the merits of the actions. The continuous actions output by the policy network are discretized into the parameters of the jamming actions in this study. The update and optimization of the network is usually performed using stochastic gradients, which can be found in more detail in [32].

### 3.2. Improved SAC Algorithmic Framework

In this paper, the case of the communication parties adjusting the communication parameters to avoid jamming is considered. At this time, the jamming party needs to learn a very large action. For example, there are four modulation modes (QPSK, BPSK, 64QAM, and 16QAM), thirty transmission powers (1, 2, 3, …, and 30), and ten transmission frequencies (1, 2, 3, 4, 5, 6, 7, 8, 9, and 10) on the communication parties. From the state space and action space defined in the previous section, the state at moment t is $S_{t}=S_{t}^{*}+a_{t}$, where $S_{t}^{*}$ is the communication parameters of the communication parties at moment t and $a_{t}$ is the jamming action of the jamming party at moment t. Then, the number of $S_{t}$ is 1,440,000 (4 × 30 × 10 × 4 × 30 × 10), and the number of jamming actions, $a_{t}$, is 1200 (4 × 30 × 10). It is assumed that the approximate parameter range of the communication parties has been obtained by the jammer through the preliminary communication reconnaissance, which means that the jamming action of the jammer and the communication state of the communication side are equal. The jamming action of the jammer is even larger than this number in practice.

According to the scenarios and problems raised above. This paper introduces an improved SAC algorithm based on the Wolpertinger architecture. The policy network outputs a continuous action space, $R^{n}$. This output is then mapped to the discrete set A. Define the function $f_{\theta^{\pi }}:S\to R^{n}$, $f_{\theta^{\pi }}\left(s\right)=\hat{a}$ to denote from the state representation space $R^{m}$ to the action representation space $R^{n}$. In this thesis, the continuous action of the SAC algorithm value network output is discretized in the following way:

(3) $$\hat{a}=\left\{round\left(a_{m}\right),round\left(a_{c}\right),round\left(a_{p}\right),\ldots ,\right\}$$

In the Equation (3), $a_{m},a_{c},\;\mathrm{and}\;a_{p}$ denote the jamming parameters output by the SAC algorithm. The round parameter denotes the rounded mathematical symbol. This operation outputs the proto-action $\hat{a}$, but the action may not be a valid action when mapping the continuous action to the discrete space, i.e., $\hat{a}\notin A$. The K-nearest neighbor (KNN) algorithm is used to solve this problem, i.e., the function $g:R^{n}\to A$ is defined, and Equation (4) returns the k actions that are most similar to the proto-action to form action set $\hat{A}$:

(4) $$g_{k}\left(\hat{a}\right)=\underset{a\in A}{\overset{k}{argmin}}|a-\hat{a}{|}_{2}$$

where a is the action in the jamming action library. In the jamming environment proposed in this paper, we assume that there is only one jammer, and only one action is executed each time. The second stage of the Wolpertinger architecture is to optimize the selection of actions by choosing the action with the highest score according to Equation (5):

(5) $$\pi_{\theta }\left(s\right)=\underset{a\in \hat{A}}{argmax}Q_{\theta^{Q}}\left(s,a\right)$$

where $Q_{\theta^{Q}}\left(s,a\right)$ is the *Q* value of the state, s, and the action, a.

**Figure 4:**
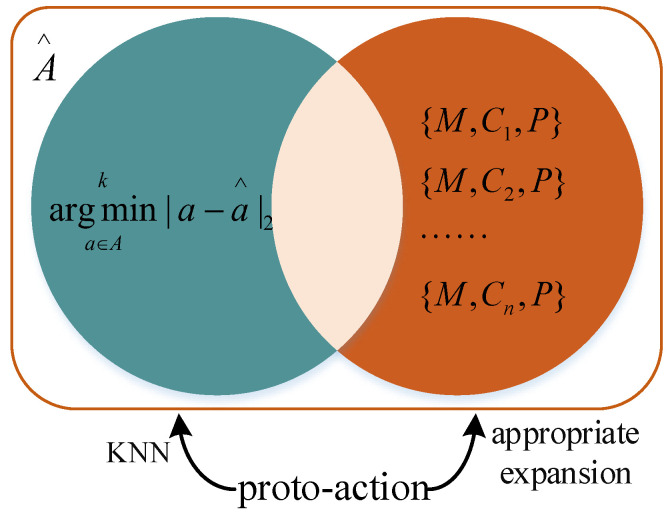
The composition of the action set, $\hat{A}$.

**Figure 5:**
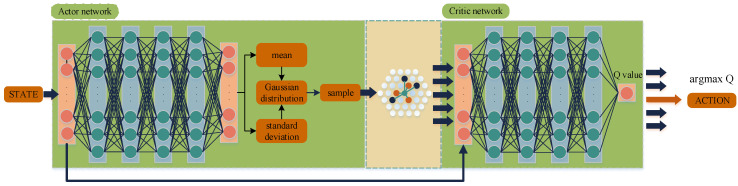
Construction of the improved SAC algorithm.

In order to speed up the convergence of the algorithm and improve the accuracy of the jamming, we propose an appropriate expansion of the set of actions, $\hat{A}$, in this paper. A large segment of the current algorithms about smart jamming are based on channel targeting because it is the prerequisite for accurate jamming. Therefore, we propose to retain the jamming modulation pattern, $\hat{M}$, and jamming power, $\hat{P}$, of the proto-action then compose them with the jamming frequencies in the jamming library into jamming actions and add these actions to action set $\hat{A\;}$, i.e., $\left\{\left(\hat{M},C_{1},\hat{P}\right),\left(\hat{M},C_{2},\hat{P}\right)\cdot \cdot \cdot \left(\hat{M},C_{n},\hat{P}\right)\right\}\to \hat{A}$. The action set, $\hat{A}$, contains two types of actions, as in Figure 4, one class for the k-nearest neighbors found by the KNN algorithm and another composed of the channel in the jamming library plus the jamming modulation, $\hat{M}$, and jamming power, $\hat{P}$, of the proto-action. Finally, the action with the largest Q value in $\hat{A}$ is selected as the actual action of the agent. Figure 5 is the main construction of the improved SAC algorithm.

### 3.3. Construction of the Network

The policy network and *Q* network of the algorithm are constructed by the fully connected layer, and the whole algorithm contains one policy network and four *Q* networks. The policy network parameter is $\varphi_{\pi }$. The four *Q* networks include two *Q* networks (*Q*_1_ network and *Q*_2_ network) and two *Q* target networks (*Q*_1_ target network and *Q*_2_ target network) whose parameters are $\varphi_{q1}$, $\varphi_{q2}$, $\varphi_{q1^{\prime }}$, and $\varphi_{q2^{\prime }}$.The use of the target network is a continuation of the fixed *Q* target strategy of the DQN algorithm. The purpose of using two *Q* networks is to solve the problem of the *Q* function overestimating the *Q* value and making the learned strategy biased. The SAC algorithm uses a pruned twin network where the *Q* value with the smaller value in the twin network is put into the value error function each time, as in Equations (6) and (7):

(6) $$q=min(Q_{1}\left(s_{t},a_{t};\varphi_{q1}\right),Q_{2}\left(s_{t},a_{t};\varphi_{q2}\right))$$

(7) $$q^{\prime }=min(Q_{1^{\prime }}\left(s_{t+1},a_{t+1};\varphi_{q1^{\prime }}\right),Q_{2^{\prime }}\left(s_{t},a_{t};\varphi_{q2^{\prime }}\right))$$

Both the policy network and the *Q* network have an input layer and an output layer. The policy network contains four hidden layers with 128, 256, 512, and 128 neurons. Each hidden layer is followed by a ReLU activation function. The activation function of the output layer is sigmoid, which limits the parameter range of the action to (0, 1). The *Q* network contains four hidden layers with 128, 256, 512, and 128 neurons, and each hidden layer is followed by a ReLU activation function. The input of the policy network is the state, $s_{t}$, and the output is the mean, μ, and the covariance, σ, of the Gaussian distribution. Then, the action and logarithm of its probability are obtained by sampling. Finally, a representation of the action, $a_{t}$, is obtained as follows:

(8) $$a_{t}\sim \pi (a_{t}|s_{t};\varphi_{\pi };\mu ;\sigma )$$

where the mean, μ, and the standard deviation, σ, of the action distribution are output from the policy network and $\pi (a_{t}|s_{t};\varphi_{\pi };\mu ;\sigma )$ denotes the policy distribution parameterized by $\varphi_{\pi }$, μ, and σ. Each dimension represents the parameters of the jamming waveform.

For the training process of the SAC algorithm, the agent learns the strategy by sampling batches of {$s_{t},a_{t},r,s_{t+1}$} from experience pool D each time. The input to the policy network is the state, $s_{t}$, and the output is policy π, which is the action distribution for $a_{t}$.

The inputs of the $Q_{1}$ network and the $Q_{2}$ network are state $s_{t}$ and action $a_{t}$, and the output dimension is 1, which represents the value of the state action ($Q_{1}$ value and $Q_{2}$ value). Similarly, the inputs to the target $Q_{1}$ network and the target $Q_{2}$ network are state $s_{t+1}$ and action $a_{t+1}$, and the output dimension is 1, which indicates the value of the state action ($Q_{1^{\prime }}$ value and $Q_{2^{\prime }}$ value). The *Q* networks are optimized using the Adam optimizer, and the *Q* network parameters are updated by minimizing the mean squared Bellman error (MSBE). The MSBE is defined as follows:

(9) $$L\left(\varphi_{q1}\right)=\frac{1}{N_{B}}\sum_{k=1}^{N_{B}}{\left(Q_{1^{\prime }}\left(s_{t+1},a_{t+1};\varphi_{q1^{\prime }}\right)-Q_{1}\left(s_{t},a_{t};\varphi_{q1}\right)\right)}^{2}$$

(10) $$L\left(\varphi_{q1}\right)=\frac{1}{N_{B}}\sum_{k=1}^{N_{B}}{\left(Q_{2^{\prime }}\left(s_{t+1},a_{t+1};\varphi_{q2^{\prime }}\right)-Q_{2}\left(s_{t},a_{t};\varphi_{q2}\right)\right)}^{2}$$

where $Q_{1^{\prime }}\left(s_{t+1},a_{t+1};\varphi_{q1^{\prime }}\right)$ denotes the target *Q* value output by the target *Q*_1_ network and $Q_{1}\left(s_{t},a_{t};\varphi_{q1}\right)$ denotes the *Q* value output by the *Q*_1_ network. $N_{B}$ denotes the size of a minibatch. $Q_{2^{\prime }}\left(s_{t+1},a_{t+1};\varphi_{q2^{\prime }}\right)$ denotes the target *Q* value output by the target *Q*_2_ network, and $Q_{2}\left(s_{t},a_{t};\varphi_{q2}\right)$ denotes the *Q* value output by the *Q*_2_ network. According to Equations (9) and (10), the parameters of the *Q*_1_ network and *Q*_2_ network can be respectively updated by:

(11) $$\varphi_{q1}=\varphi_{q1}-\beta \nabla_{\varphi_{q1}}L\left(\varphi_{q1}\right)$$

(12) $$\varphi_{q2}=\varphi_{q2}-\beta \nabla_{\varphi_{q2}}L\left(\varphi_{q2}\right)$$

where β denotes the learning rate of the *Q* network and ∇ denotes the gradient operator. The policy network parameters are updated by minimizing the Kullback–Leibler (KL) divergence, which is defined as follows:

(13) $$d\left(\varphi_{\pi }\right)=\frac{1}{N_{B}}\sum_{k=1}^{N_{B}}\left(\lambda log\pi \left(a_{t}|s_{t};\varphi_{\pi }\right)-min_{\left(i=1,2\right)}Q_{i}\left(s_{t},a_{t};\varphi_{qi}\right)\right)$$

where $\pi \left(a_{t}|s_{t};\varphi_{\pi }\right)$ denotes the policy distribution of the policy network output, $Q_{i}\left(s_{t},a_{t};\varphi_{qi}\right)$ denotes the *Q* value distribution of the $Q_{i}$ network, and λ denotes the entropy coefficient. According to Equation (13), the parameters of the policy network are updated by:

(14) $$\varphi_{\pi }=\varphi_{\pi }-\beta^{\prime }\nabla_{\varphi_{\pi }}d\left(\varphi_{\pi }\right)$$

where $\beta^{\prime }$ denotes the learning rate of the policy network and ∇ denotes the gradient operator.

**Figure 6:**
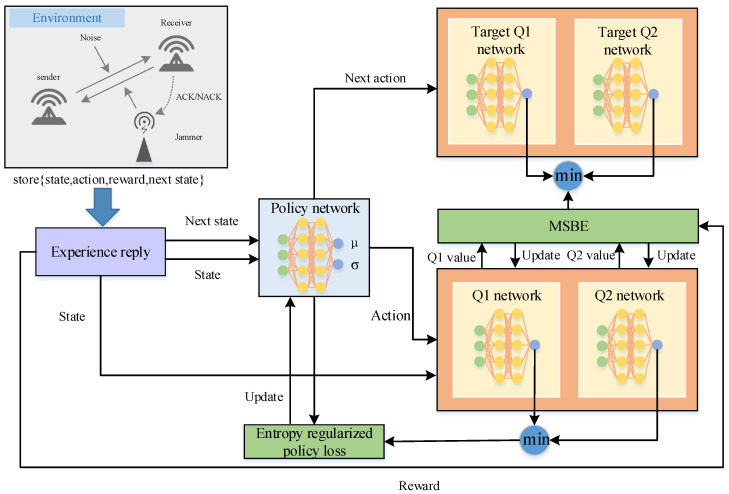
Framework of the SAC algorithm.

Figure 6 shows the framework of the SAC algorithm.

### 3.4. Overall Algorithm Flow

In summary, the proposed improved SAC algorithm adds an improved Wolpertinger architecture to the original SAC algorithm for solving complex jamming scenarios in large-scale discrete jamming spaces. Algorithm 1 gives the pseudocode of the improved SAC algorithm proposed in this paper. The output of Algorithm 1 is the parameter values of the jamming waveform, which generates the corresponding waveform to impose the jamming.
**Algorithm 1**: The proposed improved soft actor-critic algorithm.**Initialization**: Randomly initialize the parameters of the policy network and the two*Q* networks. Set the experience reply *D* with size of 100,000.**Input:** The current communication parameters of the communication parties and thecurrent jamming action of the jammer1: **for** episode *i* = 1, 2, …, J **do**2:     **for** step *j* = 1, 2, …, *N*
**do**3:          According to the state, $s_{t}$, input to the policy network sampling outputaction, $\;\;\;\;\;\;\;\;\;\;\;\;\;\;a_{t}$;4:          The proto-action, $a_{t}$, is input to the improved Wolpertinger architecture to             obtain the actual executed action, $a_{t}$;5:          Executing action $a_{t}$;6:          Obtaining the next state, $s_{t+1}$, and feedback and calculating the actualreward,              r;7:          Storing ($s_{t}$,$a_{t}$,$s_{t+1}$,r) into experience pool *D*;8:          Sampling the smallest batch, $N_{B}$, from experience pool *D* for training;9:          Updating network parameters A and B for *Q*_1_ and *Q*_2_;10:        Updating the parameters of the policy network;11:        Updating the parameters of the target *Q*_1_ and target *Q*_2_ networks;12:        Setting $s_{t}=s_{t+1}$;13:    **end for**14: **end for****Output:** Jamming action for the communication parties at the next moment

### 3.5. Computational Complexity

In the *Q*_1_ and *Q*_2_ networks, the dimensions of the input layer, the first hidden layer, the second hidden layer, the third hidden layer, the fourth hidden layer, and the output layer are, respectively, 3, *L*1, *L*2, *L*3, *L*4, and 1. In the policy network, the dimensions of the input layer, the first hidden layer, the second hidden layer, the third hidden layer, the fourth hidden layer, and the output layer are, respectively 9, *L*1, *L*2, *L*3, *L*4, and 3. The number of actions in set $\hat{A}$ is *K*. Therefore, the complexity of the improved SAC algorithm is *O*[2*K*(3*L*1 + *L*1*L*2 + *L*2 *L*3 + *L*3 *L*4 + *L*4) + 9*L*1 + *L*1*L*2 + *L*2*L*3 + *L*3*L*4 + 3*L*4]. Where *L*1, *L*2, *L*3, and *L*4 are, respectively, 128, 256, 512, and 128 and the symbol *O* represents the amount of multiplying and accumulating calculations.

## 4. Simulation Results

To demonstrate the advantages of the proposed algorithm in intelligent jamming in this paper, we designed a large number of comparative experiments, including the jamming effects in different scenarios and the effects of algorithm parameters. The experimental results show that the proposed algorithm in this paper has excellent performance in terms of the number of algorithm interactions and jamming accuracy.

### 4.1. Simulation Environment

For the experimental part, we first simulated the learning behavior of the jammer for the communication parties to switch strategies in order to resist jamming. Then, the algorithm performance of the communication parties under different switching strategies and while increasing the action space of the jammer was simulated to verify the adaptability of the algorithm. Finally, the effects of some parameters in the algorithm on the results were also simulated.

To verify the learning performance of the algorithm, we assumed that the communication parties send N symbols of data every Δt time, and if 10% of the symbols on the receiver side were incorrect, it was considered by both communication parties that the message transmission had been jammed and they needed to change the communication strategy for anti-jamming. The jamming party did not know the specific conversion method at the beginning of the period. It was assumed that the jamming party could estimate the SER of the receiver by ACK and NACK and use it as the basis for the evaluation index of the effect of jamming. The channel model was additive white Gaussian noise (AWGN), and the signal-to -noise ratio (SNR) was 20 dB for all simulations in this paper.

Our simulation environment was Matlab and PyCharm co-simulation. Matlab has powerful engine APIs that support executing Matlab commands using other programming languages without having to initiate a Matlab desktop session. PyCharm is an efficient Python IDE, and Python has the advantages of being easy to learn, supporting multiple deep learning frameworks, and being portable. These make it feasible to use the advantages of both Matlab and Python to implement decision simulations of communication intelligence jamming algorithms. The Matlab side was responsible for communication signal generation, modulation, Gaussian channel transmission, demodulation, filtering, SER calculation, conversion strategy, and other steps related to communication signals. The PyCharm side was responsible for the overall design of the algorithm proposed in this paper, the decision making of jamming actions, data processing, environmental reconnaissance, and other steps related to the decision algorithm steps. The parameter settings in the proposed algorithm are shown in Table 1. The learning rate parameters β and $\beta^{*}$ in Table 1 were adjusted to 0.003 when the jamming action space was 20. The adjustment of the entropy coefficient is discussed in detail in Section 4.2.7.

### 4.2. Comparative Experiment

In this subsection, we compare the jamming effects of the improved SAC algorithm, the SAC algorithm, the classic DQN algorithm, and the Q-learning algorithm adopted in [28] under different jamming scenarios. The DDPG algorithm performed poorly or even failed to train in each of the environments proposed in this paper. Therefore, the results are not shown in this paper. It was verified that the algorithm proposed in this paper has adaptability in different scenarios.

#### 4.2.1. The Number of Jamming Actions Was 150

We assumed that the anti-jamming method of the communication parties was to change the communication parameters. The communication parameters included the modulation mode, transmit power, and transmission frequency. Specifically, there were three modulation modes (QPSK, BPSK, and FSK), five transmission powers (1, 2, 3, 4, and 5), and ten transmission frequencies (1, 2, 3, 4, 5, 6, 7, 8, 9, and 10). The anti-jamming method of the communication parties was as follows:

A. The initial communication parameters were that the modulation mode was QPSK, the transmission power was 1, and the communication frequency was 5. If the receiver SER exceeded 10%, they turned to procedure B.
B. The transmit power was increased. If the increase in the maximum transmit power still exceeded the threshold of SER, they turned to procedure C.
C. The communication frequency was switched according to the rule of frequency points (5, 6, 9, 10, 4, 7, 1, 3, 8, and 2), and each new frequency point chose the minimum transmit power until this frequency point to increase the maximum transmit power, which was still jammed. Then, it was considered that this frequency point was always jammed when the signal was transmitted using QPSK modulation. If there was still jamming after all frequencies had been switched, they turned to procedure D.
D. The communicator switched the modulation mode and repeated the above procedure in the new modulation mode.

**Figure 7:**
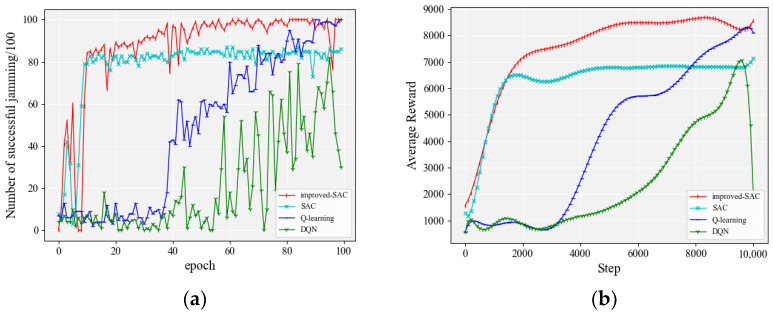
Performance comparison of each algorithm when the number of jamming actions was 150: (**a**) accuracy of jamming; (**b**) smoothed average reward.

The performance of each algorithm with 150 jamming actions is shown in Figure 7. Table 2 gives some details of the data during the experiment. The three columns on the right side of the table represent the number of rounds where the jamming accuracy exceeded 80% for the first time, the number of rounds where the jamming accuracy exceeded 90% for the first time, and the average jamming accuracy after the accuracy exceeded 80%.

The algorithm parameters of Q-learning and DQN are, respectively, shown in Table 3 and Table 4, where i denotes the number of training rounds and *j* denotes the number of interactions per round. The network structure of DQN is consistent with the parameters of the policy network in the SAC algorithm. The network inputs and outputs of DQN are consistent with the inputs and outputs of the policy network in the SAC algorithm. The size of Q-form in Q-learning is the number of states, $N_{s}$, multiplied by the number of actions, $N_{a}$. The exploration utilization factor of the DQN algorithm is as follows:

(15) $$1-\left(0.01+C_{1}\times \exp\left(-1\times j\times i\times C_{2}\right)\right)$$

where exp represents the exponential notation in mathematics. *i* and *j* are the same as defined in the previous paragraph. $C_{1}$ is a constant equal to 0.98. $C_{2}$ is the attenuation coefficient. A large $C_{2}$ value is set when the jamming action space is large, and a small $C_{2}$ value is set when the jamming action space is small. The $C_{2}$ value set in this paper was taken as the value with the best result in the experiment. Equation (15) represents an exponential form of the plot, indicating that more exploration was given at the beginning of the jamming process and more exploitation was given at the end of the jamming process.

It can be seen from Figure 7a that, among the compared algorithms, the SAC-based algorithm converged faster and reached 80% accuracy quickly. The Q-learning algorithm [28] and the DQN algorithm took a long time to explore the environment when the jamming space was 150. In terms of accuracy, the improved SAC algorithm exceeded 90% jamming accuracy for the first time in the 29th round and continued to improve. It reached 100% accuracy for the first time in the 60th round and mostly stayed above 98% accuracy after that. The original SAC algorithm was not able to make any significant breakthrough after reaching an accuracy of 80%. From the previous section, it is clear that the goal of an agent in reinforcement learning is to pursue higher rewards. Figure 7b shows the smoothed average rewards of the agent during training. It can be seen that the rewards of the agent in the improved SAC algorithm in this paper all exceed or are equal to the other comparison algorithms for the same number of training steps.

#### 4.2.2. The Number of Jamming Actions Was 600

**Figure 8:**
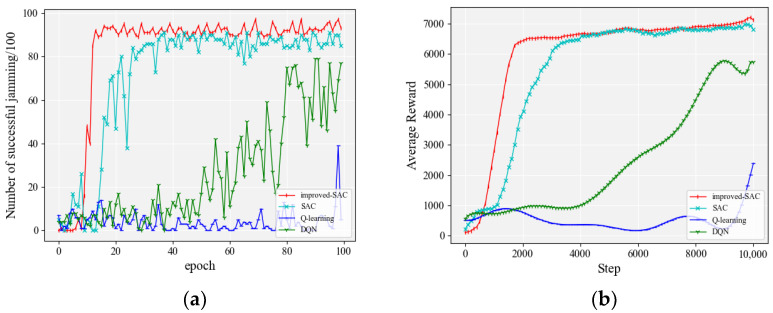
Performance comparison of each algorithm when the number of jamming actions was 600: (**a**) accuracy of jamming; (**b**) smoothed average reward.

We increased the number of jamming actions, which assumed that there were three modulation modes (QPSK, BPSK, and FSK), ten transmission powers (1, 2, 3, 4, 5, 6, 7, 8, 9, and 10), and twenty transmission frequencies (1, 2, 3, 4, 5, 6, 7, 8, 9, 10, 11, 12, 13, 14, 15, 16, 17, 18, 19, and 20). The anti-jamming method for both sides of the communication followed the method in Section 4.2.1, which meant that the anti-jamming proceeded by way of increasing the power first, then switching the communication frequency points, and finally adjusting the communication mode. The frequency point started from the first one for each new modulation mode, and the transmitting power started from the minimum for each new frequency point. The frequency switching mode of the communication parties was 16, 3, 8, 2, 19, 15, 10, 12, 11, 14, 4, 1, 6, 7, 9, 5, 20, 18, 13, and 17. The initial communication parameters were as follows: the modulation mode was QPSK, the transmission power was 1, and the communication frequency was 16. Figure 8 shows a comparison of the jamming effects of each algorithm. In order to visualize the performance of different algorithms, detailed accuracy data are given in Table 5.

It can be seen from Figure 8a that, when the number of actions increased to 600, Q-learning [28] was no longer able to perfect the Q-form in 100 rounds of interaction. DQN showed a trend of convergence by virtue of the excellent fitting ability of the neural network, but it still required a longer time to explore. The convergence speed based on the SAC algorithm was significantly better than the other algorithms. The improved SAC algorithm not only improved the convergence speed compared to the original SAC algorithm but also improved the accuracy rate by 7.7%, as shown in Table 5. At the same time, Figure 8b also clearly shows that the improved SAC algorithm proposed in this paper had a faster convergence speed.

#### 4.2.3. The Number of Jamming Actions Was 1200

**Figure 9:**
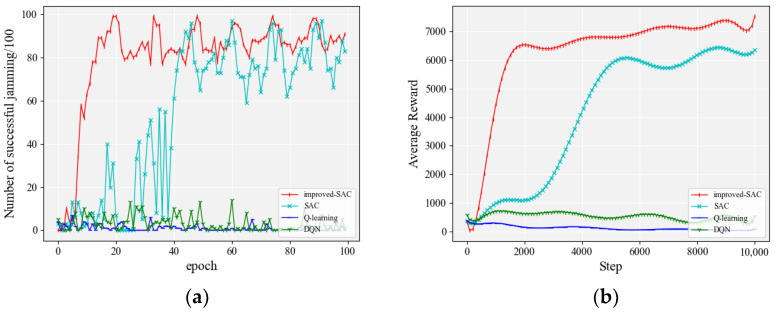
Performance comparison of each algorithm when the number of jamming actions was 1200: (**a**) accuracy of jamming; (**b**) smoothed average reward.

We continued to increase the number of jamming actions to 1200, which assumed that there were three modulation modes (QPSK, BPSK, and FSK), ten transmission powers (1, 2, 3, 4, 5, 6, 7, 8, 9, and 10), and forty transmission frequencies (1, 2, 3, 4, …, and 40). The anti-jamming mode of the communication parties followed the method in Section 4.2.1. The frequency switching mode of the communication parties was 3, 38, 9, 35, 24, 1, 23, 12, 30, 6, 19, 25, 17, 36, 33, 7, 10, 16, 37, 40, 8, 4, 31, 22, 2, 21, 11, 28, 29, 39, 18, 5, 32, 13, 15, 26, 27, 20, 14, and 34. The initial communication parameters were as follows: the modulation mode was QPSK, the transmission power was 1, and the communication frequency was 3. Figure 9 shows a comparison of the jamming effects of each algorithm. In order to visualize the performance of different algorithms, detailed accuracy data are given in Table 6.

It can be seen from Figure 9a that the improved SAC algorithm had a much higher convergence speed than other algorithms for 1200 actions. It is worth mentioning that both the SAC algorithm and the improved SAC algorithm in the figure exhibited regular sawtooth waveforms. This is due to the fact that when the number of actions increased to 1200 there were 40 cases of channel conversion, and not all conversion strategies could be fully learned within 100 rounds. The regular sawtooth waveforms in the figure have a period of about 12, which corresponds to exactly 1200 actions, and it takes 12 rounds to execute all these actions. The improved SAC algorithm also showed a slow upward trend in zigzagging. The original SAC algorithm also started to converge after 40 rounds, but it was slower and the accuracy was not as high as the improved SAC algorithm in the later stages. The DQN and Q-learning algorithms [28] could not converge in the first 100 rounds of training with 1200 actions, which indicates that DQN and Q-learning are not suitable for handling large-scale action spaces. Figure 9b indicates that the improved SAC algorithm in this paper showed a more obvious advantage in terms of both convergence speed and average reward when the space of jamming actions of the agent reached 1200.

#### 4.2.4. The Number of Jamming Actions Was 20

**Figure 10:**
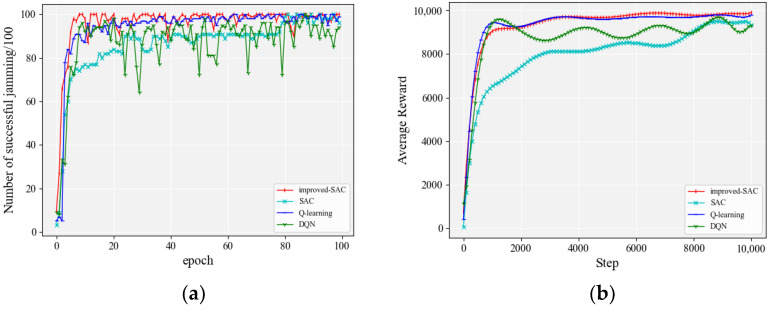
Performance comparison of each algorithm when the number of jamming actions was 20: (**a**) accuracy of jamming; (**b**) smoothed average reward.

The current jamming models about communication parties changing the communication parameters after jamming have many assumptions that the communication parties have fewer conversions. Therefore, the case where the number of jamming actions was 20 was also simulated, which meant that there were two modulation modes (QPSK and BPSK), two transmission powers (1 and 2), and five transmission frequencies (1, 2, 3, 4, and 5). The anti-jamming mode of the communication parties followed the method in Section 4.2.1. The frequency switching mode of the communication parties was 2, 1, 3, 5, and 4. The initial communication parameters were as follows: the modulation mode was QPSK, the transmit power was 1, and the communication frequency point was 2. Figure 10 shows a comparison of the jamming effects of each algorithm. In order to visualize the performance of different algorithms, detailed accuracy data are given in Table 7.

It can be seen from Figure 10a that both Q-learning [28] and DQN were better in this situation with a small number of actions. In particular, Q-learning took a short time to build the Q table and had high stability when there was a finite number of states and actions. It is worth mentioning that the improved SAC algorithm showed even better performance than Q-learning in the scenario of 20 action spaces. It exceeded 90% accuracy in the 6th round of training and reached 100% accuracy in the 9th round. This shows that the algorithm proposed in this paper has excellent performance, even in the case of small action spaces. Significantly, Figure 10b shows that the accuracy and sliding average reward curves are not uniform. The improved SAC algorithm proposed in this paper performed best in terms of accuracy, but the Q-learning algorithm performed best in terms of average rewards. The difference between the improved SAC algorithm and Q-learning in terms of jamming accuracy was not very large. However, due to the power penalty factor and channel reward factor set in the rewards, the improved SAC algorithm quickly learned the power conversion method, while the Q-learning algorithm had a higher average probability of predicting channel switching accurately, making its overall average reward greater than that of the improved SAC algorithm. The DQN algorithm also performed well in the early rewards but was unstable in the later stages.

#### 4.2.5. Selecting 20 Jamming Actions from the Jamming Library

**Figure 11:**
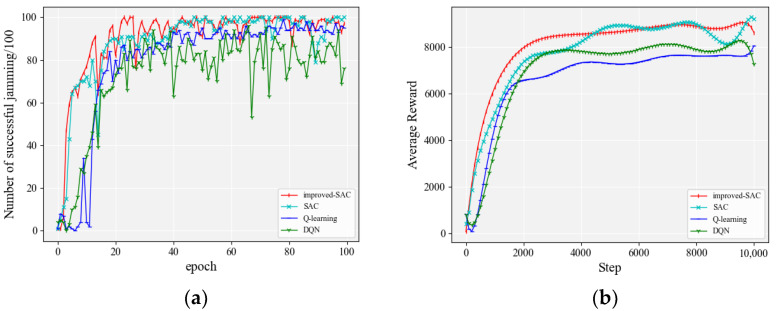
Performance comparison of each algorithm when selecting 20 actions from the action library: (**a**) accuracy of jamming; (**b**) smoothed average reward.

In practice, the jammer in many cases does not know the exact range of anti-jamming parameters of the communication parties, which means that the number of jamming actions and the number of communication parameters of the communication parties are unequal. In this case, the jammer needs to select actions from the jamming library to perform jamming and learn the anti-jamming strategy of the communication parties. Therefore, the case of 20 switching strategies for both communication sides when the jamming action library was 600 was simulated. The frequency switching mode of the communication parties was 2, 1, 3, 5, and 4, and the other conversion parameters remained the same as in Section 4.2.4. The waveform parameters of the jamming action library were composed of modulation modes (QPSK, BPSK, and FSK), transmit powers (1~10), and communication frequencies (1~20). Figure 11 shows a comparison of the jamming effects of the different algorithms. In order to visualize the performance of different algorithms, detailed accuracy data are given in Table 8.

As can be seen from Figure 11a, it took longer to explore to select the jamming action from the action library and learn its conversion strategy than to learn the conversion strategy directly when the size of the action library was 600. Compared with Figure 10a, it can be seen that the convergence speed was slower for the same scenario and training parameters. All four algorithms in the figure could converge in less than 100 rounds, while the improved SAC algorithm converged the fastest and achieved 100% accuracy for the first time in round 24. The original SAC algorithm reached 100% accuracy for the first time in round 48. The average reward curve in Figure 11b is consistent with the accuracy curve.

#### 4.2.6. Selecting 150 Jamming Actions from the Jamming Library

**Figure 12:**
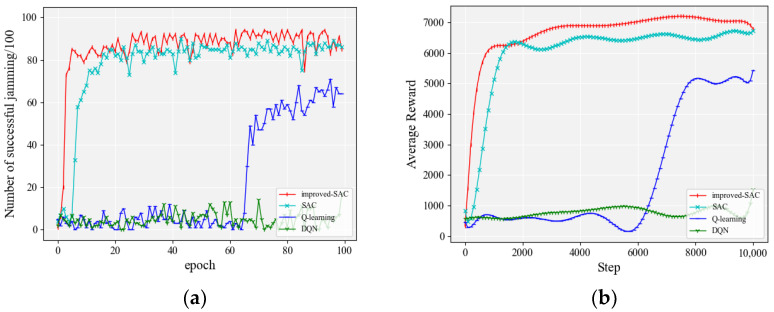
Performance comparison of each algorithm when selecting 150 actions from the action library: (**a**) accuracy of jamming; (**b**) smoothed average reward.

We increased the number of conversion strategies to 150 for both communication parties based on Section 4.2.5. The frequency switching mode of the communication parties was 16, 1, 9, 3, 13, 18, 8, 7, 4, 5. Figure 12 shows a comparison of the jamming effects of the different algorithms. In order to visualize the performance of different algorithms, detailed accuracy data are given in Table 9.

From Figure 12a, it can be seen that the accuracy of each algorithm decreased when the jamming action range was extended to 150. However, the algorithm proposed in this paper had the fastest convergence speed and the highest accuracy rate among the compared algorithms. This conclusion is also reflected in Figure 12b.

#### 4.2.7. The Effect of Temperature/Entropy Coefficient

As mentioned in the previous section, the entropy coefficient controls the randomness of the optimal strategy. The inclusion of the entropy coefficient not only encourages exploration but also allows the agent to learn the near-optimal behavior. The larger the entropy coefficient, the more the agent explores the environment. In [34], the authors proposed an automatic adjustment of the entropy coefficient, which means that more exploration should be given in the interval of action uncertainty. In the non-cooperative environment proposed in this paper, the jammer does not know the strategy transformation pattern of the communication parties in the initial stage of jamming. The jammer can only figure out the conversion strategy of the communication parties by randomly selecting the jamming parameters. Therefore, more exploration is needed by the jammer in the early stage of jamming. The jammer continuously learns from its historical experience as the number of jams increases. At this time, it should make full use of the environmental information already learned and give the algorithm a smaller entropy factor. In this paper, we refer to the strategy of the automatic adjustment of entropy coefficients in [34] and use the following formula for the strategy adjustment:

(16) J(α)=1−log(ceil((eps+1)/100),10)×0.5

where eps represents the number of training rounds, ceil represents the upward rounding function, and log is the logarithmic function.

**Figure 13:**
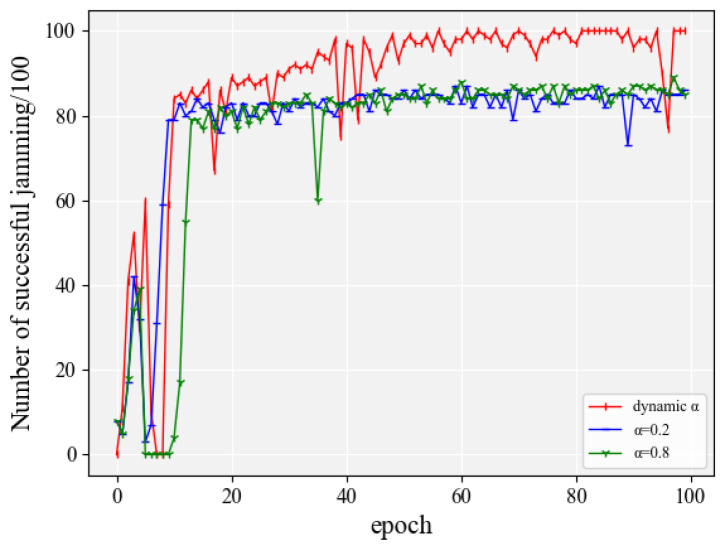
The effect of the entropy coefficient on algorithm performance when the action space was 150.

As can be seen from Figure 13, in the scenario proposed in this paper, it should be based on different exploration probabilities in different periods to better improve the jamming accuracy and save jamming resources. Therefore, the effect is not as effective as that of dynamic entropy coefficients when setting fixed entropy coefficients. From Figure 13, it can be seen that the jamming accuracy of the jammer does not increase after reaching a certain value when the entropy coefficient is set to 0.2 and 0.8.

#### 4.2.8. The Effect of Discount Factor γ

**Figure 14:**
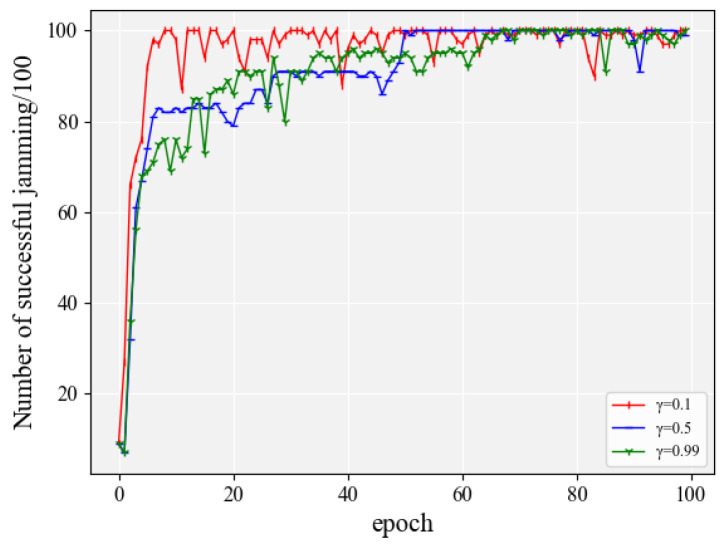
The effect of discount factor γ when the action space was 20.

In most cases of algorithms for reinforcement learning, the agent is required to consider long-term rewards. If γ = 0, the agent focuses only on maximizing the timely reward, which means that the goal of the agent is to maximize $R_{t+1}$ [33]. In the scenario proposed in this paper, the jammer gives a jamming action for the next moment based on the current communication condition, and we want the jammer to be able to successfully jam at each step, which means that the reward for each step is real and significant. There is no state where the reward is much higher than the other rewards according to the rewards covered in this paper. Therefore, the impact of different discount factors on the convergence performance of the algorithm was simulated. As shown in Figure 14, the algorithm converged fastest when γ = 0.1, followed by γ = 0.5, and finally γ = 0.99. The speed of convergence of the algorithm has been given more attention than the long-term cumulative reward. This is because in a real battlefield environment it is important to acquire more experience in fewer interactions. Therefore, the discount rate, γ, in the algorithms designed in this paper are all equal to 0.1.

### 4.3. Discussion

In this section, we discuss the effect of the algorithm proposed in this paper in different jamming scenarios. The initial intention of our proposed algorithm was to solve intelligent jamming problems in complex scenarios. In particular, when the situation is complex, ordinary reinforcement learning algorithms require a high number of interactions or even fail to converge. The experiments demonstrated that the improved SAC algorithm showed better performance than other intelligent algorithms in several scenarios proposed in this paper. They showed that the maximum-entropy-based reinforcement learning algorithm can effectively balance the dilemma of exploration and exploitation in reinforcement learning. The proposed algorithm is more trusting of the value network than the general actor-critic framework. This is due to the fact that in the scenario proposed in this paper it was more difficult to accurately yield the parameters of the jamming waveform than to evaluate the value of the action. This provides a new perspective for subsequent research on reinforcement learning. We also found in our experiments that the proposed algorithm outperformed ordinary reinforcement learning algorithms, even in the case of small action spaces. However, the present algorithm has shortcomings that will be addressed in future work:

1. The parameters in the jamming action library will be further extended so that it can cope with more complex communication jamming scenarios;
2. The case where the channel is occupied will be taken into account, i.e., the scenario where the communication parties choose the channel to communicate after negotiation. Intelligent algorithms will incorporate algorithms such as case-based reasoning to avoid unnecessary exploration and further accelerate the convergence of algorithms;
3. In this paper, the use of SER as a reward was not optimal, and more realistic and simple rewards will be tried in the future. On the basis of maintaining the original model, the model structure will be further improved to enhance the robustness of the system.

## 5. Conclusions

In this paper, we propose a cognitive electronic jamming decision model based on an improved SAC for the intelligent jamming decision system. The Wolpertinger architecture for solving large-scale discrete action spaces was applied to the SAC algorithm so that the algorithm proposed in this paper showed good performance in scenarios with both small and large action spaces. We designed numerous comparative experiments to demonstrate the excellent performance of the proposed algorithm. Compared with ordinary reinforcement learning algorithms, the proposed algorithm in this paper was improved in terms of convergence speed and accuracy. The penalty factor of reward and the dynamic entropy coefficient were also designed to further optimize the structure of the algorithm in this paper. The algorithm proposed in this paper greatly reduces the number of interactions of the jammer and uses effective jamming resources to achieve accurate jamming. A new solution is provided for the field of intelligent communication jamming.

## Figures and Tables

**Table 1:** Hyperparameters for the SAC-based algorithm.

Description	Value
Learning rate of actor network	β=0.0015 $\beta^{*}=0.0015$
Learning rate of critic network	
Size of experience pool	*D* = 10^6^
Return discount factor	γ=0.1
Learning rate of soft update	τ=0.005
Training batch size	$N_{B}=100$
Entropy coefficient	Dynamic

**Table 2:** Detailed data comparison of the performance of each algorithm when the jamming action space was 150.

Method	Accuracy		
	≥80% (First)	≥80% (First)	Average (≥80%)
Improved-SAC	11	29	93.85%
SAC	12	\	83.26%
Q-learning	61	81	85.25%
DQN	95	\	52.4%

**Table 3:** Q-learning algorithm parameters.

Description	Value
Update Steps	0.9 γ=0.1
Discount Factor	
Explore–Exploit Factor	Ceil (i/10)/10

**Table 4:** DQN algorithm parameters.

Description	Value
Discount Factor	γ=0.1
Learning rate	0.003
Batch Size	$N_{B}=100$
Experience Pool Size	*D* = 10^6^
Explore–Exploit Factor	Dynamic

**Table 5:** Detailed data comparison of the performance of each algorithm when the jamming action space was 600.

Method	Accuracy		
	≥80% (First)	≥80% (First)	Average (≥80%)
Improved-SAC	13	14	91.95%
SAC	23	37	85.34%
Q-learning	\	\	\
DQN	\	\	\

**Table 6:** Detailed data comparison of the performance of each algorithm when the jamming action space was 1200.

Method	Accuracy		
	≥80% (First)	≥80% (First)	Average (≥80%)
Improved-SAC	15	18	87.83%
SAC	43	45	78.83%
Q-learning	\	\	\
DQN	\	\	\

**Table 7:** Detailed data comparison of the performance of each algorithm when the jamming action space was 20.

Method	Accuracy		
	≥80% (First)	≥80% (First)	Average (≥80%)
Improved-SAC	6	9	98.29%
SAC	16	82	90.99%
Q-learning	5	63	96.69%
DQN	13	56	93.70%

**Table 8:** Detailed data comparison of the performance of each algorithm when 20 actions were selected from the jamming action library.

Method	Accuracy		
	≥80% (First)	≥80% (First)	Average (≥80%)
Improved-SAC	12	24	95.31%
SAC	13	48	93.65%
Q-learning	19	\	90.59%
DQN	24	\	82.18%

**Table 9:** Detailed data comparison of the performance of each algorithm when 150 actions were selected from the jamming action library.

Method	Accuracy		
	≥80% (First)	≥80% (First)	Average (≥80%)
Improved-SAC	6	22	88.36%
SAC	17	44	84.37%
Q-learning	\	\	\
DQN	\	\	\

## Data Availability

Not applicable.
